# The circadian-microbiome-immune axis in colorectal cancer: mechanisms of spatiotemporal immunosuppression and chronotherapeutic horizons

**DOI:** 10.3389/fimmu.2026.1925490

**Published:** 2026-08-10

**Authors:** Liangchen Li, Guiying Wang

**Affiliations:** 1 Department of General Surgery, The Second Hospital of Hebei Medical University, Shijiazhuang, Hebei, China; 2 Hebei Key Laboratory of Etiology Tracing and Individualized Diagnosis and Treatment for Digestive System Carcinoma, The Second Hospital of Hebei Medical University, Shijiazhuang, Hebei, China

**Keywords:** chronotherapy, circadian rhythm, colorectal cancer, gut microbiome, immunotherapy resistance, mucosal barrier, myeloid-derived suppressor cells, taurocholic acid

## Abstract

Colorectal cancer (CRC) remains a major global oncological challenge, largely because most microsatellite-stable (MSS) tumors exhibit intrinsic resistance to immune checkpoint blockade (ICB). Emerging evidence suggests that the gut microbiome and host circadian rhythm jointly regulate mucosal homeostasis; however, their dynamic integration—defined here as the circadian-microbiome-immune axis—remains incompletely understood in CRC. In this review, we synthesize preclinical findings and the limited available clinical evidence concerning this axis. Experimental studies using genetic and lifestyle-related models of circadian disruption indicate that clock dysfunction may impair the intestinal mucosal barrier and alter microbial composition, including depletion of beneficial taxa such as *Lactobacillus*. These changes have been associated with metabolic remodeling, including increased taurocholic acid (TCA), which has been shown in cell and animal models to activate the RasGRF1/MAPK/ERK pathway. Preclinical evidence further suggests that intestinal clock disruption may interact with Wnt signaling and chemokine networks, including CXCL5 and IL-17, thereby promoting the rhythmic recruitment of PD-L1-expressing myeloid-derived suppressor cells (MDSCs). On this basis, we propose the Temporal Immunosuppressive Shield (TIS) as a hypothesis-generating conceptual framework linking epithelial barrier dysfunction, microbial metabolic remodeling, and temporally gated myeloid-mediated suppression of cytotoxic CD8^+^ T cells. Because this integrated model has not yet been directly validated in human MSS CRC, it should not be regarded as an established biological mechanism. We also discuss the potential of biologically phase-aligned ICB and chemotherapy, conceptually termed chronoimmunotherapy, while emphasizing current challenges in circadian biomarker development, interindividual variability, and mouse-to-human translation. Overall, this review outlines a testable spatiotemporal framework and identifies the experimental and clinical studies required to evaluate its relevance to therapeutic resistance in CRC.

## 1 Introduction: the spatiotemporal nexus of time, microbes, and colorectal cancer

Colorectal cancer (CRC) remains one of the most prevalent and lethal malignancies worldwide, imposing a substantial global burden on public health (1). Although immune checkpoint blockade (ICB) targeting the PD-1/PD-L1 pathway has transformed the treatment of metastatic MSI-H/dMMR CRC, its benefit in the broader CRC population remains limited (2, 3). In contrast, pMMR/MSS CRC has shown little activity with PD-1 blockade as monotherapy (4). pMMR/MSS CRC is frequently characterized by a non-inflamed tumor microenvironment with limited cytotoxic T-cell infiltration, which is one potential barrier to response to immune checkpoint blockade (5). Defining the mechanisms that limit antitumor immunity in pMMR/MSS CRC is therefore an important objective for improving immunotherapy.

Recently, mucosal homeostasis has been recognized as a dynamic process shaped by reciprocal interactions between the gut microbiome and the host circadian clock (6, 7). At the cellular level, the biological clock is an evolutionarily conserved timekeeping system driven by transcriptional-translational feedback loops, primarily governed by transcription factors CLOCK and BMAL1 (8, 9). Within the gastrointestinal tract, circadian clocks regulate epithelial, stem-cell, and immune-cell programs, although the evidence for each process derives from distinct experimental systems (10–13). Concurrently, the gut microbiome interacts with the intestinal mucosa and immune system to help maintain homeostasis (14). Daily oscillations in microbial composition and metabolic output have been documented in experimental models, although their contribution to human intestinal barrier function remains incompletely defined (6, 7).

Current evidence suggests that the host circadian clock and the gut microbiota participate in a bidirectional homeostatic relationship, referred to here as the circadian-microbiome-immune axis (6, 7). Experimental studies show reciprocal associations between host circadian rhythms, microbial composition, and host metabolic transcriptional programs (7, 15). Genetic epithelial-clock disruption and sleep deprivation have each been associated with impaired intestinal homeostasis and CRC-related phenotypes in experimental models (11, 16). Independent studies have shown that myeloid and other immune cells possess cell-intrinsic circadian programs that regulate inflammatory responses (12, 13), while the intestinal mucus barrier is central to maintaining host–microbiota compartmentalization (17). Preclinical CRC studies associate epithelial-clock disruption with barrier dysfunction and microbial dysbiosis (11). In mouse CRC models, PD-L1-expressing MDSCs exhibit rhythmic accumulation and suppress CD8^+^ T-cell activity, supporting a potential contribution to ICB resistance; however, this mechanism has not yet been directly validated in human MSS CRC (18).

In this review, we synthesize these emerging observations through the proposed Temporal Immunosuppressive Shield (TIS) framework. Existing host clock-microbiome models have primarily emphasized the circadian regulation of microbial oscillations and metabolic homeostasis (6, 7), whereas microbiome-immunity frameworks have focused on microbial determinants of inflammatory tone and therapeutic responsiveness. More recently, Liu et al. provided mechanistic evidence in mouse models that circadian dysfunction can promote colorectal cancer metastasis through gut microbiota-dependent accumulation of myeloid-derived suppressor cells, with taurocholic acid acting as a key mediator (19). The same study also reported clinical associations among circadian disruption, increased myeloid-cell accumulation, and metastatic disease (19). However, this study primarily addressed systemic metastatic dissemination and did not conceptualize epithelial barrier disruption, microbial metabolic remodeling, and temporally gated immune exclusion as a spatially organized barrier within the colorectal tumor microenvironment.

Accordingly, the TIS differs from existing host clock-microbiome models by focusing on a CRC-specific, spatially organized hypothesis in which epithelial barrier impairment, microbiota-associated metabolic remodeling, and temporally varying myeloid-mediated immune exclusion may converge in pMMR/MSS tumors. This framing complements, rather than replaces, broader bacteria-based therapeutic concepts and microbiome-immunity frameworks (20).

**Figure 1:**
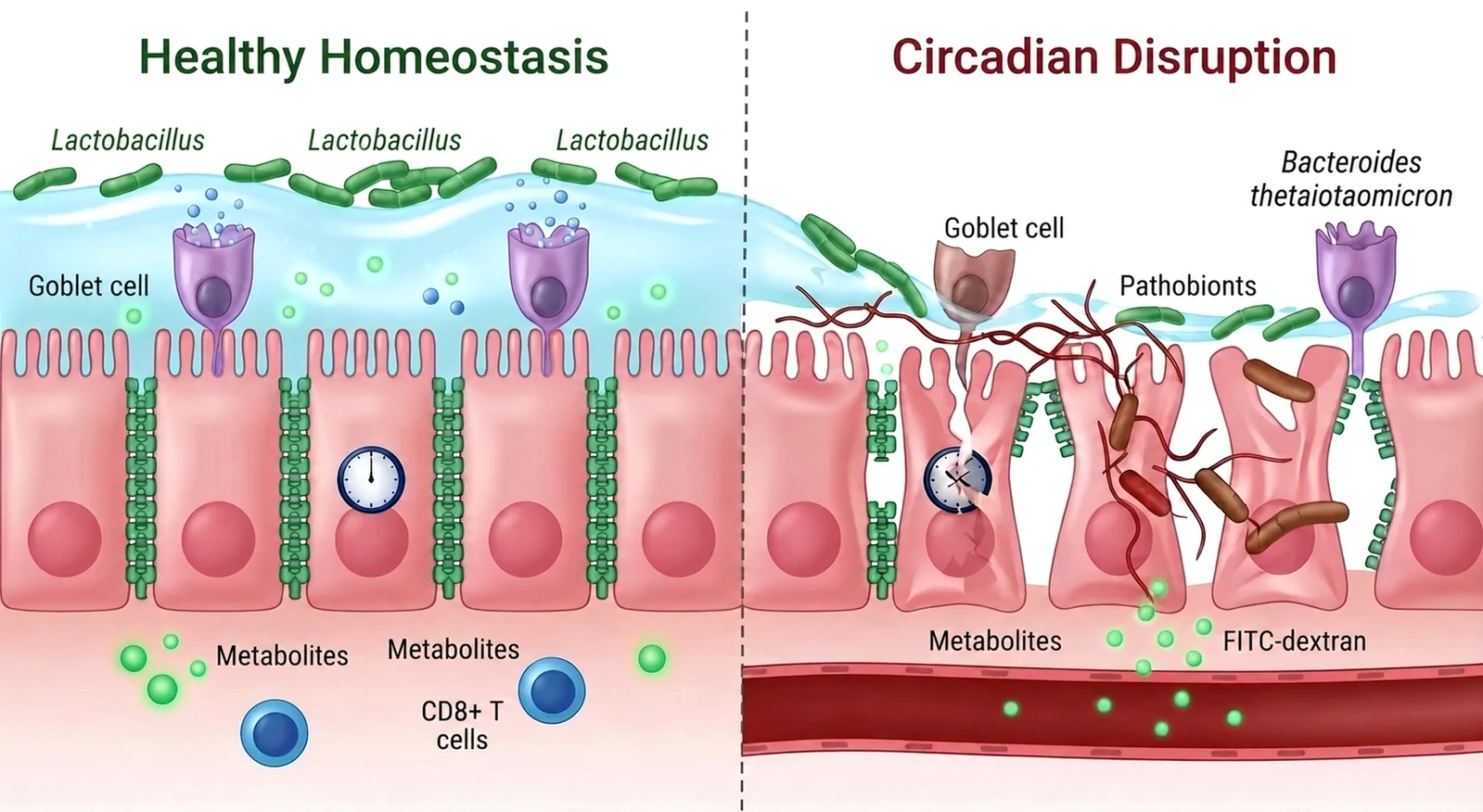
Proposed effects of circadian disruption on the intestinal mucosal barrier and microbiome in colorectal cancer. The left panel depicts homeostatic mucus and tight-junction organization, whereas the right panel summarizes changes reported in epithelial clock-disrupted mouse models and synchronized epithelial cell systems, including reduced mucin and tight-junction gene expression, increased permeability, and altered microbial composition. Solid arrows denote experimentally supported relationships; dashed arrows denote proposed or incompletely validated connections; and faded elements indicate impaired pathways. The lower modules illustrate epithelial clock regulation, junction impairment, and potential mucin utilization by *Bacteroides thetaiotaomicron*. The integrated sequence is based mainly on preclinical evidence and has not been validated as a unified mechanism in human CRC. This image was created by the authors based on published evidence, principally Refs (11, 17, 21, 32).

Building on this evidence, we propose the TIS as a hypothesis-generating conceptual framework that links three interdependent layers: epithelial barrier dysfunction as a potential spatial entry point, microbiota-associated metabolic remodeling as a biochemical relay, and temporally varying myeloid accumulation with CD8^+^ T-cell suppression as a candidate effector layer. The TIS should not be interpreted as an independently validated biological entity or as a replacement for existing circadian-microbiome models. Rather, it organizes observations from preclinical studies and limited clinical associations into a set of testable predictions (11, 18, 19). In the sections that follow, we examine evidence that circadian disruption may impair epithelial barrier integrity and reshape microbial composition (6, 11, 21), discuss candidate metabolic pathways including TCA and RASGRF1/MAPK/ERK signaling (16), and evaluate preclinical data linking epithelial clock disruption to rhythmic myeloid accumulation and altered CD8^+^ T-cell activity (18, 19). We also consider the investigational potential of phase-aligned therapy while emphasizing the current lack of validated human biomarkers, prospective CRC-specific trials, and direct evidence that the complete TIS sequence operates in patients. Accordingly, this review is intended to define experimentally addressable questions rather than to present the circadian-microbiome-immune axis as an established mechanism of therapeutic resistance in CRC (**Table 1**, **Figure 1**).

**Table 1:** Evidence-informed comparison of features relevant to the circadian-microbiome-immune axis across intestinal states.

Feature	Healthy mucosa	dMMR/MSI-H colorectal cancer	pMMR/MSS colorectal cancer
Circadian Evidence	Rhythmic epithelial and microbiota-associated functions demonstrated mainly in experimental models (6, 7, 10, 15)	CRC-subtype-specific circadian alterations remain insufficiently characterized	Clock disruption and associated phenotypes demonstrated primarily in mouse CRC models; direct human validation remains limited (11, 18)
Mucosal Barrier	Intact mucus and tight-junction organization under physiological conditions (17, 21, 23)	No consistent subtype-specific clock-related barrier phenotype has been established	Mucin loss and altered tight-junction regulation reported in clock-disrupted mouse models (11)
Gut Microbiome	Diverse microbial communities with diurnal taxonomic and functional oscillations in experimental studies (6, 7, 15)	Heterogeneous; no universal dMMR/MSI-H-specific circadian microbial signature has been established	Model-dependent dysbiosis, including shifts in *Bacteroidetes* and selected taxa; generalizability to human MSS CRC remains uncertain (11, 16)
Microbial Metabolites	Rhythmic SCFAs and microbiota-associated metabolic signaling reported mainly in experimental models (6, 38)	Insufficient evidence for a subtype-specific circadian metabolite profile	Increased fecal TCA and reduced *Lactobacillus* abundance reported in sleep-disrupted mouse models (16)
Immune Microenvironment	Homeostatic mucosal immune surveillance (14)	Lymphocyte-rich and immunologically active tumors are more frequently observed (5)	Frequently immune-excluded and poorly responsive to ICB, although substantial interpatient heterogeneity exists (5, 48)
Rhythmic Myeloid Suppression	Physiological circadian regulation of innate immune cells; tumor-associated MDSC rhythmicity is not applicable (12, 13)	Rhythmic PD-L1-expressing MDSC accumulation has not been established in human dMMR/MSI-H CRC	Rhythmic Gr-1^+^ PD-L1^+^ myeloid-cell accumulation reported in mouse tumor models; not established in human MSS CRC (18)
Response to ICB	Not applicable	Clinically meaningful responses to PD-1 blockade occur in many patients (2–4)	Response to current ICB is generally limited, with important interpatient heterogeneity (5, 48)

Findings related to circadian regulation, the microbiome, microbial metabolites, and myeloid cells are derived predominantly from preclinical studies unless otherwise specified. A lack of subtype-specific evidence should not be interpreted as evidence of no biological effect.

## 2 Intestinal clock disruption and gut dysbiosis in CRC: evidence from mucosal barrier models

### 2.1 Intestinal epithelial *Bmal1* ablation and mucosal barrier dysfunction

BMAL1 (brain and muscle ARNT-like 1; encoded by *ARNTL*) is a core transcriptional regulator of the molecular circadian clock. Together with CLOCK, BMAL1 drives rhythmic transcriptional programs that support cell-autonomous timekeeping (8, 9). Here, the term “epithelial clock” refers to the cell-autonomous molecular circadian timing system operating within intestinal epithelial cells, rather than the central circadian pacemaker or whole-organism sleep–wake rhythms (8, 9, 11). This local timing system contributes to the regulation of mucin-associated and tight-junction-associated genes and thereby helps maintain epithelial barrier homeostasis (11). Epithelial-specific *Bmal1* deletion using the Villin-Cre system is therefore used experimentally to disrupt the local intestinal epithelial clock and to examine how loss of epithelial circadian organization affects mucus production, junctional integrity, and paracellular permeability in CRC-related models (11, 22). In the Apc-driven CRC model cited here, *Apc* and *Bmal1* were selectively deleted in intestinal epithelial cells, whereas peripheral clocks remained intact; this design therefore examines the local epithelial-clock contribution rather than whole-body circadian disruption (11, 18).

The intestinal mucus layer forms a major biophysical barrier that limits direct contact between luminal microorganisms and the underlying epithelium (17). In healthy intestinal mucosa, gel-forming mucin 2, encoded by *Muc2*, and transmembrane mucins such as mucin 3, encoded by *Muc3*, contribute to mucus production and barrier maintenance (21, 23). In *Apc^+^/^-^* mouse models with Villin-Cre-mediated intestinal epithelial deletion of *Bmal1*, reduced expression of several mucin-associated genes, including *Muc2*, *Muc3*, *Muc3a*, and *Muc13*, was reported (11, 24). Histological analysis using Periodic acid–Schiff staining showed fewer goblet cells and reduced mucus thickness in advanced tumor regions in these models (11). These findings support a preclinical association between epithelial clock loss and impaired mucus-barrier integrity. However, whether these alterations directly initiate colorectal tumorigenesis or occur to the same extent in human CRC remains uncertain (11, 21).

The epithelial tight-junction complex contributes to the regulation of paracellular permeability and limits the translocation of luminal antigens (25). Junction-associated proteins include occludin, encoded by *Ocln*, and zonula occludens-1 (ZO-1), encoded by *Tjp1 (*25, 26). In *Apc*-deficient mouse models with intestinal epithelial deletion of *Bmal1*, transcriptomic analyses identified reduced expression of several junction-associated genes, including *Ocln*, *Cldn7*, *Cldn15*, and *Tjp1 (*11). An increase in *Cldn8* expression was also observed, potentially reflecting a compensatory response rather than preservation of overall barrier function (11). Circadian variation in tight-junction components has been reported in other epithelial tissues (27, 28), but the relevance of these observations to human CRC remains uncertain. Collectively, the available evidence supports an association between epithelial clock disruption and altered tight-junction regulation in preclinical models, without establishing an equivalent mechanism in patients.

Complementary evidence from synchronized human Caco-2 monolayers suggests that barrier-related genes are subject to cell-autonomous circadian regulation, although this *in vitro* system should not be considered equivalent to clinical validation (11). In synchronized cultures, core clock genes, including *BMAL1* and *NR1D1*, and junction-associated genes, including *OCLN* and *CLDN8*, exhibit rhythmic expression (11). Experimental clock disruption in these monolayers, as well as epithelial *Bmal1* loss in *Apc*-driven mouse models, was associated with increased paracellular permeability to 4-kDa fluorescein isothiocyanate (FITC)–dextran (11). Environmental circadian-disruption studies in rodents have additionally reported alterations in intestinal physiology and gut microbial composition (29, 30). Collectively, these findings support a preclinical role for circadian regulation in epithelial barrier integrity; however, direct validation in human CRC tissues and prospective patient cohorts remains lacking.

### 2.2 Barrier dysfunction and clock-associated microbial dysbiosis in CRC

Barrier disruption may create conditions that favor alterations in microbial community structure, although the directionality of this relationship remains incompletely resolved (11, 31). In tumor-bearing, clock-deficient *Apc^+^/^-^*; *Bmal1^-^/^-^* mice, metagenomic profiling identified a reduction in the relative abundance of *Firmicutes* and a corresponding expansion of *Bacteroidetes* compared with control animals (11, 31). These findings indicate that epithelial clock loss is associated with phylum-level dysbiosis in this preclinical setting. However, phylum-level shifts are taxonomically broad and may vary according to diet, housing conditions, sequencing methods, and baseline microbial composition; therefore, they should not be interpreted as a universal microbial signature of human CRC. Whether these compositional changes contribute causally to barrier dysfunction, inflammation, or tumor progression requires strain-level and functional validation.

At the species level, multivariable analyses in clock-deficient mouse models of CRC identified increased relative abundance of several taxa, including *Bacteroides acidifaciens*, *Bacteroides caecimuri*, *Bacteroides thetaiotaomicron*, and *Helicobacter apodemus (*11). These observations indicate an association between epithelial clock disruption and enrichment of selected microbial species in this preclinical setting, but they do not establish that these taxa are pathogenic or causally involved in CRC progression. *Bacteroides thetaiotaomicron* has the capacity to utilize host-derived mucin glycans when dietary polysaccharides are limited (32). Under conditions of pre-existing barrier impairment, this mucin-foraging activity could potentially contribute to further thinning of the mucus layer. However, whether this process materially increases intestinal permeability or microbial translocation in clock-disrupted CRC models has not been directly demonstrated. Accordingly, the proposed feed-forward interaction between barrier dysfunction and mucin-utilizing bacteria should be regarded as a biologically plausible but incompletely validated mechanism (11, 31, 32).

In the same preclinical study, clock disruption was also associated with increased relative abundance of several additional taxa, including *Fusobacterium mortiferum*, *Parasutterella excrementihominis*, and *Paramuribaculum intestinale* (11). In the cited study, sleep deprivation in *Apc*^Min/+^ mice was induced using a modified multiple-platform paradigm consisting of 24 h of deprivation followed by 48 h of recovery, repeated over a 12-week period (16). Thus, the animals were exposed to the deprivation condition during one-third (33.3%) of each 72-h cycle; however, actual sleep duration was not objectively recorded, and the percentage reduction relative to normal sleep time was not reported (16). This 33.3% value therefore describes the experimental exposure schedule rather than a directly measured percentage of sleep loss. In the cited study, *Lactobacillus* was identified as the genus showing the most pronounced reduction in relative abundance in sleep-deprived mice. However, the published 16S rRNA analysis did not resolve or report a specific *Lactobacillus* species or strain (16). These observations suggest a shift in microbial community structure and function, but the biological significance of individual taxa remains uncertain. The effects of *Lactobacillus* are likely to be strain- and context-dependent, and current evidence does not establish that its depletion directly impairs CD8^+^ T-cell activity or promotes CRC progression in humans. Accordingly, these taxonomic changes should be interpreted as model-dependent associations that require validation through strain-resolved sequencing, metabolite profiling, and functional intervention studies.

Beyond the taxa discussed above, CRC immunotherapy studies have reported associations between ICI response and several commensal groups, including *Akkermansia*, *Bifidobacterium*, and *Faecalibacterium*. However, the available CRC evidence is limited in number and heterogeneous in treatment regimen, cohort composition, and sequencing strategy; it therefore cannot establish any single taxon as a causal determinant of response (33). These organisms should be regarded as candidate microbial correlates rather than universal biomarkers. At a functional level, SCFAs, indole derivatives, and bile acids provide plausible routes through which microbial ecology could influence epithelial and immune states; however, their circadian dynamics, tissue exposure, and disease specificity in pMMR/MSS CRC remain insufficiently defined (34–36).

## 3 Preclinical evidence for circadian disruption-associated metabolic remodeling: a candidate TCA–RASGRF1–MAPK/ERK pathway

Metabolomic profiling in sleep-deprived *Apc^Min/+^* mice identified changes in the fecal metabolite profile, including enrichment of pathways related to taurine and hypotaurine metabolism (16). Taurocholic acid (TCA) was among the metabolites increased in fecal samples, and this difference was further assessed using enzyme-linked immunosorbent assay (16). In this mouse model, fecal TCA levels were inversely associated with the relative abundance of *Lactobacillus (*16). Because some *Lactobacillus* strains possess bile salt hydrolase activity, reduced abundance of these bacteria could plausibly contribute to altered conjugated bile acid metabolism (16, 36). However, the relative contributions of microbial bile salt hydrolase activity, host bile acid synthesis, intestinal transit, and other microbial functions remain unresolved. These findings support a preclinical association between sleep disruption, microbiome alterations, and increased fecal TCA, but they do not establish that this pathway occurs uniformly in human CRC or that TCA is the dominant mediator of circadian disruption-associated tumor progression.

Experimental studies have examined the potential tumor-promoting effects of elevated TCA in preclinical CRC models. In human CRC cell lines, including HCT116 and SW480 cells, exogenous TCA treatment was associated with increased cell viability, clonogenic growth, migration, and invasive capacity (16). TCA exposure also reduced the expression of the junction-associated proteins ZO-1 and E-cadherin in these experimental systems, suggesting a potential contribution to epithelial barrier dysfunction (16). In nude mice bearing HCT116 xenografts, systemic TCA administration was associated with increased tumor volume and tumor weight (16). Related *in vivo* evidence further suggests that alterations in bile acid exposure may influence CRC progression (37). Collectively, these findings support a tumor-promoting role for TCA in preclinical models; however, the physiologically relevant concentrations, pharmacokinetics, and tissue exposure of TCA in human CRC remain insufficiently defined.

Beyond TCA, microbial metabolites including short-chain fatty acids (SCFAs) exhibit diurnal variation in experimental models (38). Dietary and microbial perturbations may alter these rhythms, but their relevance to CRC remains uncertain (39). SCFAs can influence epithelial and immune-cell function through histone deacetylase inhibition and G-protein-coupled receptor signaling in experimental systems (34). However, direct evidence linking circadian disruption, altered SCFA availability, and CRC progression in humans remains limited. Accordingly, SCFA-associated immune remodeling should be regarded as a biologically plausible component of the circadian-microbiome-immune axis rather than an established mechanism in human CRC.

Microbiota-associated tryptophan metabolism represents another potential link between circadian disruption and mucosal immunity. Dietary tryptophan availability and microbial community composition can influence microbial tryptophan metabolism and the generation of indole derivatives, including indole-3-propionic acid and indole-3-lactic acid, although the responsible pathways are species- and strain-dependent (35, 40). These metabolites may engage the aryl hydrocarbon receptor (AhR) in epithelial and immune cells and thereby influence barrier-related transcription and IL-22-associated mucosal responses in experimental systems (35, 41). Some *Lactobacillus* strains can contribute to indole metabolism, but reduced *Lactobacillus* abundance should not be assumed to produce a uniform loss of AhR signaling across hosts. Moreover, direct evidence that circadian disruption alters the temporal production of these metabolites in CRC, or that such alterations drive myeloid recruitment and immunotherapy resistance in humans, remains lacking. The tryptophan–AhR–IL-22 pathway should therefore be considered a biologically plausible but incompletely validated component of the proposed circadian-microbiome-immune axis.

**Figure 2:**
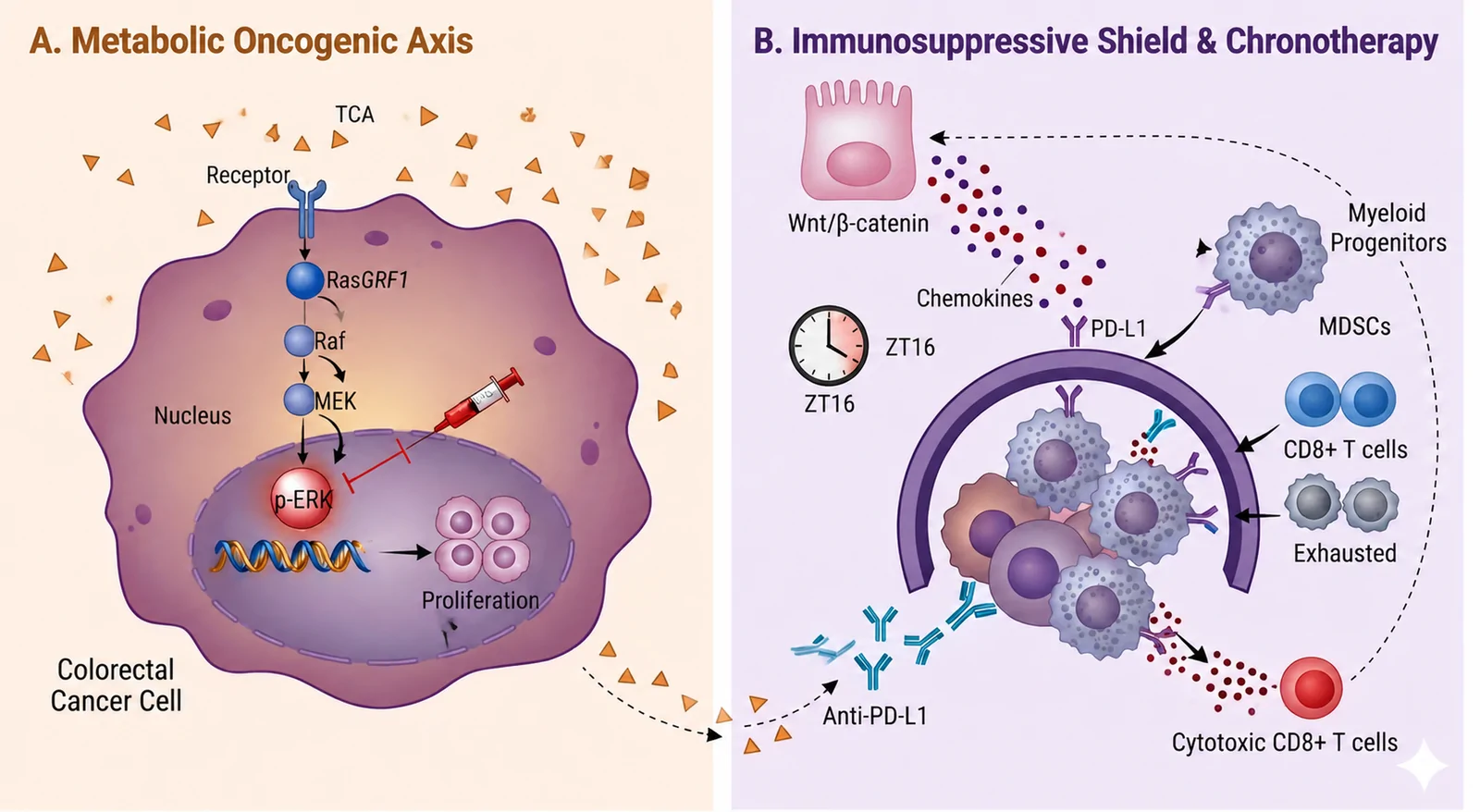
Proposed metabolic and immune components of the temporal immunosuppressive shield framework. **(A)** Metabolic oncogenic axis: sleep disruption in preclinical models is associated with reduced Lactobacillus abundance, increased fecal taurocholic acid (TCA), and candidate RASGRF1–MAPK/ERK signaling. **(B)** Immunosuppressive shield and chronotherapy: epithelial clock disruption in mouse tumor models is associated with altered Wnt/chemokine signaling, rhythmic Gr-1^+^ PD-L1^+^ myeloid-cell accumulation, and reduced CD8^+^ T-cell activity. Solid arrows indicate relationships supported in experimental systems; dashed arrows indicate hypothetical feedback or translational links; and red bars indicate experimental blockade. Anti-PD-L1 treatment at ZT16 showed greater activity than treatment at ZT4 in selected mouse models, but no corresponding optimal treatment window has been established in human CRC. This image was created by the authors based on published evidence, principally Refs (16, 18, 19).

Mechanistic studies in TCA-treated CRC cells have implicated MAPK signaling as a candidate downstream pathway (16). Transcriptomic profiling and Gene Set Enrichment Analysis (GSEA) identified Ras protein-specific guanine nucleotide-releasing factor 1 (*RASGRF1*) as a potential upstream regulator of the Ras–RAF–MEK–ERK cascade (16). Supporting evidence from other tumor contexts indicates that RASGRF1 can regulate Ras/MAPK activity, while MAPK signaling is a well-established contributor to CRC progression (42, 43). In these experimental systems, TCA exposure was accompanied by increased RASGRF1 expression and ERK phosphorylation (16). Pharmacological inhibition of ERK with SCH772984 or genetic silencing of *RASGRF1* using small hairpin RNA attenuated TCA-associated cell proliferation, migration, and invasion (16). These findings support a preclinical TCA–RASGRF1–MAPK/ERK signaling axis; however, its concentration dependence, tumor-context specificity, and validity in human CRC specimens remain to be established (**Figure 2**).

Taken together, the studies summarized in this section do not establish that circadian disruption causes CRC development through TCA, SCFAs, or tryptophan-derived metabolites in humans. Most of the mechanistic evidence currently derives from CRC cell lines, mouse models, or other experimental systems without direct validation in patients with CRC (16, 34, 35, 38–41). Collectively, the cited studies do not provide integrated, time-resolved measurements of circulating, fecal, and intratumoral metabolite concentrations, pharmacokinetics, or tissue exposure in patients with CRC (16, 34–41). Moreover, the available human observations have not demonstrated the complete causal sequence from circadian disruption to metabolite remodeling, myeloid cell recruitment, and immunotherapy resistance (18, 19). Accordingly, the TCA–RASGRF1–MAPK/ERK axis and the other metabolite pathways discussed here should be interpreted as candidate preclinical mechanisms and priorities for future validation, rather than as established determinants of CRC initiation, progression, or treatment response (16, 18, 19, 34–41).

## 4 Preclinical evidence for rhythmic myeloid-mediated immunosuppression in CRC

Preclinical evidence suggests that circadian disruption-associated metabolic remodeling may occur alongside changes in local immune signaling. In *Apc*-driven mouse models and intestinal epithelial systems, epithelial-clock disruption was associated with altered Wnt/β-catenin signaling and increased expression of selected chemokines and cytokines (11, 18). Multiplex cytokine profiling identified CXCL5, IL-5, CCL5, and IL-17 among the factors altered following epithelial *Bmal1* loss (18). Among these mediators, CXCL5 has been proposed as a candidate clock-sensitive chemoattractant. Collectively, these findings support the possibility that epithelial clock loss and Wnt dysregulation cooperate to generate a chemotactic environment favoring the recruitment of neutrophilic and monocytic myeloid cells into the colorectal tumor microenvironment (18). However, the relative contribution of each chemokine and the extent to which this pathway operates in human MSS CRC remain uncertain.

In *Apc*-driven, clock-disrupted mouse models, recruited myeloid cells acquire transcriptional and functional features consistent with an immunosuppressive MDSC phenotype (11, 18, 44). Single-cell RNA sequencing of CD45^+^ tumor-infiltrating immune cells identified a myeloid cluster enriched for suppressive-associated genes, including *S100a8, S100a9, Wfdc17, and Arg2 (*18). These markers have been linked to immature myeloid states and immunosuppressive activity: S100A8 and S100A9 may support the accumulation and maintenance of MDSC-like populations, whereas arginase activity, reactive oxygen species, and PD-L1 expression represent established mechanisms through which myeloid cells can impair T-cell responses (44–47). In coculture assays, Gr-1^+^ myeloid cells isolated from these mouse models inhibited CD8^+^ T-cell proliferation and granzyme production in a dose-dependent manner (18). These findings support the presence of a functionally suppressive myeloid population in preclinical CRC models; however, they do not establish that identical cellular subsets or suppressive mechanisms operate in human MSS CRC.

Preclinical evidence further indicates that the abundance and suppressive activity of tumor-associated myeloid cells may vary according to circadian phase (18). In mouse intestinal tumor models, Gr-1^+^ PD-L1^+^ myeloid cells exhibited rhythmic accumulation, with higher abundance reported during the early active phase at Zeitgeber Time 16 (ZT16) (18). By contrast, epithelial clock disruption in *Apc^+^/^-^; Bmal1^-^/^-^* mice was associated with loss of this rhythmic pattern and persistently elevated myeloid-cell accumulation (18). These observations support the existence of a potential chrono-gated window of immunosuppression in preclinical CRC models. During phases of increased accumulation of PD-L1-expressing myeloid cells, reduced CD8^+^ T-cell activity may facilitate tumor immune escape (18). However, the temporal profile, cellular composition, and clinical relevance of this phenomenon have not yet been established in human MSS CRC (**Figure 2**).

MDSCs are only one component of the immunosuppressive CRC microenvironment. Regulatory T cells, tumor-associated macrophages, tolerogenic dendritic cells, and natural killer (NK) cells can also shape effector-cell recruitment or activity in CRC (48); however, their direct integration into a circadian-microbiome axis has not been established. Circadian programs have been described in macrophages and dendritic cells in experimental systems, but CRC-specific studies that jointly measure clock state, microbial features, and these immune populations remain scarce (12, 13). Thus, this review treats MDSCs as the best-developed example in the current evidence base, rather than as the sole immunosuppressive effector population.

## 5 Translational horizons: shaping the chronoimmunotherapy of colorectal cancer

Preclinical studies suggest that the timing of anti-PD-L1 administration may influence treatment response when tumor-associated myeloid populations vary across the circadian cycle (18). In *Apc^+^/^-^; Bmal1^-^/^-^* mouse models, anti-PD-L1 was administered twice weekly at either ZT4 or ZT16 to evaluate this phase-dependent hypothesis (18). Treatment at ZT16 was associated with lower accumulation of tumor-infiltrating Gr-1^+^ MDSCs and greater CD8^+^ T-cell infiltration than treatment at ZT4 (18). Over the three-week treatment period, neither schedule significantly altered the total polyp number, whereas a reduction in polyp size was observed in the ZT16-treated group (18). These findings provide preclinical evidence that treatment timing may modify anti-PD-L1 activity, but they do not establish an optimal dosing schedule for patients with CRC. Observational associations between immune checkpoint inhibitor infusion timing and clinical outcomes have been reported in other cancers, including melanoma; however, prospective CRC-specific validation remains lacking (49).

Additional preclinical evidence was obtained using syngeneic subcutaneous MC38 tumor models (18). In this experimental setting, anti-PD-L1 treatment administered at ZT16 was associated with complete responses in 4 of 14 tumors (29%), compared with 1 of 12 tumors (8%) treated at ZT4 (18). Progressive disease occurred in 4 of 14 tumors (29%) in the ZT16 group and 6 of 12 tumors (50%) in the ZT4 group (18). Although these numerical differences are consistent with a potential time-of-day effect on treatment response, the modest sample sizes and use of a murine subcutaneous tumor model limit direct clinical interpretation. These findings should therefore be regarded as hypothesis-supporting preclinical evidence rather than proof that a corresponding optimal treatment window exists in patients with CRC.

Translating these findings to patients will require individualized circadian phase assessment rather than direct conversion of murine ZT16 to a fixed clock time in humans (18). Candidate phase markers for future study include dim-light melatonin onset (DLMO), sleep-wake timing, and serial peripheral-blood measurement of clock-gene transcripts such as *PER2* and *BMAL1*; however, their reproducibility and predictive value for CRC treatment scheduling remain uncertain (50). Observational studies in melanoma have reported associations between immune checkpoint inhibitor infusion timing and clinical outcomes (49, 51), whereas chronomodulated chemotherapy has been evaluated in patients with CRC (52, 53). These findings do not establish a specific morning or afternoon window for ICB in CRC. Prospective studies should therefore compare biomarker-defined circadian phases and prespecified dosing windows before individualized chronoimmunotherapy schedules are recommended.

Preclinical evidence suggests that circadian disruption may influence chemotherapy sensitivity through microbiome-associated metabolic changes described above (16). In sleep-deprived CRC mouse models, fecal microbiota transplantation or supplementation with *Lactobacillus* partially restored 5-FU sensitivity, but the responsible microbial functions and contribution of TCA-related signaling remain unresolved (16). Related studies have shown that diet and feeding patterns can reshape the diurnal dynamics of the gut microbiome (54), supporting the broader plausibility of microbiome-based circadian modulation. However, the responsible microbial functions, durability of the treatment effect, safety of microbiome interventions, and relevance to human CRC have not been established. Therefore, combining microbiome modulation with chronotherapy should currently be regarded as an experimental strategy that requires mechanistic validation and prospective clinical evaluation (**Table 2**, **Figure 2**).

**Table 2:** Selected experimental and clinical evidence relevant to the circadian-microbiome-immune axis in colorectal cancer.

Study focus	Experimental or clinical model	Key observation	Interpretation and evidence boundary	References
Intestinal Clock–Microbiota Homeostasis	Conventional and germ-free mouse models under controlled light–dark conditions	Diurnal microbial oscillations were associated with rhythmic epithelial and metabolic programs	Supports bidirectional clock–microbiota regulation mainly in non-tumor preclinical systems	(6, 7, 15, 58)
Epithelial Clock Disruption in CRC	Villin-Cre-mediated intestinal epithelial deletion of *Bmal1* in *Apc^+^/^-^* mouse models	Reduced mucin- and tight-junction-associated gene expression, increased permeability, and altered microbial composition	Provides preclinical evidence linking epithelial clock loss to barrier dysfunction; equivalent mechanisms have not been established in human CRC	(11)
Sleep Disruption and TCA-Associated Signaling	Sleep-deprived *Apc^Min/+^* mice, CRC cell lines, and xenograft models	Reduced *Lactobacillus* abundance, increased fecal TCA, and candidate RASGRF1–MAPK/ERK signaling	Supports a preclinical metabolic pathway; physiologically relevant TCA exposure and human applicability remain uncertain	(16)
Rhythmic Myeloid Immunosuppression	*Apc^+^/^-^*; *Bmal1^-^/^-^* intestinal tumor models and syngeneic MC38 tumors	Rhythmic Gr-1^+^ PD-L1^+^ myeloid-cell accumulation and phase-dependent anti-PD-L1 activity	Demonstrated in mouse models; corresponding cellular rhythms and optimal treatment timing have not been established in human CRC	(18)
Circadian Dysfunction and Metastatic Progression	Mouse cancer models with complementary clinical association analyses	Gut microbiota-dependent MDSC accumulation and TCA-associated signaling were linked to metastatic progression	Includes mechanistic mouse evidence and associative human findings, but does not validate the complete TIS framework in patients	(19)
Microbiome Modulation and Chemotherapy Sensitivity	Sleep-disrupted *Apc^Min/+^* models treated with fecal microbiota transplantation or *Lactobacillus* supplementation	Microbiome intervention partially restored 5-FU sensitivity in experimental models	Preclinical evidence only; durability, safety, responsible strains, and clinical efficacy remain unknown	(16)
ICB Infusion Timing in Humans	Observational cohorts of patients with advanced melanoma receiving immune checkpoint inhibitors	Infusion timing was associated with differences in clinical outcomes	Non-randomized and non-CRC evidence; these studies cannot define an optimal ICB schedule for CRC	(49, 51)
Chronomodulated Chemotherapy in CRC	Randomized studies comparing chronomodulated and conventional delivery of oxaliplatin, fluorouracil, and folinic acid in metastatic CRC	Timing-based chemotherapy schedules were clinically evaluated	Supports the feasibility of chemotherapy chronomodulation but does not establish an optimal immunotherapy schedule or validate the TIS framework	(52, 53)

Evidence concerning epithelial clock disruption, microbial remodeling, TCA-associated signaling, and rhythmic myeloid suppression is derived predominantly from preclinical models. Human studies of treatment timing are limited to observational immune checkpoint inhibitor studies in non-CRC populations and clinical trials of chronomodulated chemotherapy in CRC. These findings should not be interpreted as validation of a unified TIS mechanism or a clinically established treatment window.

### 5.1 Translational blueprint for future clinical studies

To evaluate whether circadian phase influences therapeutic response in patients with pMMR/MSS metastatic CRC, a prospective biomarker-informed trial could compare prespecified dosing windows without assuming that a specific human clock time is optimal (18, 53). Baseline assessment could include dim-light melatonin onset (DLMO), sleep-wake timing, and serial peripheral-blood measurement of clock-gene transcripts such as *PER2* and *BMAL1*, although assay feasibility, reproducibility, and patient burden would require preliminary validation (50). Participants could then be randomized to standard scheduling or a phase-aligned arm defined by individualized circadian markers rather than by direct translation of murine Zeitgeber Time values. Anti-PD-L1 and fluoropyrimidine-based treatment should be evaluated separately or within carefully designed combination cohorts because current evidence does not establish that the same circadian phase is optimal for immunotherapy and chemotherapy (16, 18, 52, 53). Potential clinical endpoints could include safety, treatment adherence, objective response rate, and progression-free survival, accompanied by exploratory analyses of CD8^+^ T-cell infiltration, circulating myeloid populations, and microbiome-associated metabolic profiles. Such a design should be regarded as a hypothesis-generating framework rather than a ready-to-implement clinical protocol and would require pilot feasibility testing before formal phase II evaluation.

## 6 Critical knowledge gaps and future directions: bridging the chrono-microbiome divide

### 6.1 Genetic clock ablation versus environmental sleep disruption: mechanistic divergence

Clarifying the distinction between genetic clock ablation and environmental circadian disruption is important for interpreting preclinical evidence concerning the circadian-microbiome-immune axis (16, 18, 55). Intestinal epithelial-specific deletion of *Bmal1* using Villin-Cre in *Apc*-driven mouse models provides a tractable approach for examining the local functions of the epithelial clock (11, 22, 55). In these experimental systems, epithelial clock loss has been associated with altered Wnt signaling, mucin depletion, barrier dysfunction, and chemokine expression (11, 18). However, these models do not exclude potential contributions from systemic physiology, developmental compensation, or interactions with other peripheral clocks. By contrast, chronic sleep deprivation and shift-work paradigms perturb central and peripheral circadian organization and may affect neuroendocrine, metabolic, and immune pathways (55, 56). Genetic and environmental models should therefore be regarded as complementary rather than equivalent, and future studies should directly compare their effects on epithelial integrity, microbial function, myeloid-cell phenotypes, and CD8^+^ T-cell activity (16, 18).

Across experimental studies, the direction and magnitude of circadian-disruption-associated changes in microbial composition and antitumor immunity are not uniform (16, 18, 29, 30). Outcomes can vary according to the disruption paradigm, host genotype, diet, housing, sampling phase, tumor model, and analytical workflow; apparent differences should therefore not be interpreted as evidence for a common microbial signature or a fixed treatment-timing effect in human CRC. Future studies should standardize reporting of light-dark conditions, feeding schedules, sampling phase, and microbiome methods, and should directly compare genetic and environmental perturbations within matched CRC models.

### 6.2 The nocturnal-to-diurnal translational bottleneck in chronotherapeutic translation

Translation of circadian treatment effects from nocturnal rodents to diurnal humans is complicated by species differences in activity phase, feeding behavior, endocrine rhythms, and tumor biology (16, 18, 50). In mouse CRC models, ZT16 represents the early active phase under a standard light-dark cycle, but it should not be interpreted as a direct human clock-time equivalent (18, 50, 56). Candidate markers for future studies include dim-light melatonin onset, cortisol phase, sleep-wake timing, and serial peripheral-blood measurement of clock-gene transcripts such as *PER1*, *PER2*, and *BMAL1*; however, their feasibility, reproducibility, and clinical relevance remain uncertain (50). Future clinical studies should therefore align treatment with measured biological phase rather than assume that mouse ZT16 corresponds to a uniform morning or afternoon window in humans (50). Repeated sampling of circulating immune cells and, when feasible, tumor tissue could be used to determine whether temporally varying myeloid or T-cell phenotypes are present in human MSS CRC (12, 18, 50). Until such evidence becomes available, chronoimmunotherapy schedules derived from mouse models should remain investigational.

### 6.3 Establishing causality in clock-modulated microbiota

Current studies demonstrate associations between circadian disruption, altered microbial composition, and impaired intestinal barrier function, but they do not establish whether the identified microbial taxa are causal drivers, downstream consequences, or context-dependent correlates of CRC progression (11). Addressing this uncertainty will require functional experiments that move beyond taxonomic profiling. Germ-free or gnotobiotic mouse models colonized with defined microbial communities or candidate strains could be used to test whether clock-associated microorganisms alter tumor burden, barrier integrity, metabolite production, and immune-cell composition (11, 57). Such studies should incorporate strain-level characterization, physiologically relevant microbial exposure, appropriate dietary controls, and microbial depletion or rescue experiments to distinguish direct microbial effects from host-mediated changes. Parallel validation using human-derived intestinal or tumor organoid systems and prospective patient cohorts will also be required before specific microorganisms are considered therapeutic targets or clinical biomarkers.

### 6.4 Characterizing the bidirectional host-microbiome circadian loop

Current models frequently describe the circadian-microbiome-immune axis as a predominantly unidirectional sequence in which host circadian disruption contributes to barrier dysfunction, microbial dysbiosis, metabolic remodeling, and altered antitumor immunity (11, 16, 18, 58). However, experimental evidence from non-CRC settings suggests that microbial oscillations and microbiota-derived metabolites may also influence host transcriptional and circadian programs (15, 58). Whether specific metabolites altered during circadian disruption, including TCA, SCFAs, and tryptophan derivatives, can directly modify the expression or rhythmicity of clock genes such as *BMAL1*, *CLOCK*, and *NR1D1* in colorectal epithelial cells or tumor-infiltrating immune cells remains uncertain. It is also unknown whether such effects are sufficient to establish a self-reinforcing feedback loop in human CRC. Future studies integrating time-resolved metabolomics, transcriptomics, microbial profiling, and histologic or immune-context measurements, together with functional perturbation experiments, will be required to determine the directionality, strength, and disease relevance of these interactions (15, 59). Multimodal integration of microbiome, transcriptomic, and histologic data has improved survival-risk stratification across several cancer types (60). Its utility for circadian phase assignment or treatment scheduling in CRC remains untested. Until these relationships are experimentally demonstrated, the proposed bidirectional loop should be regarded as a testable hypothesis rather than an established mechanism.

Emerging regulatory processes, including liquid-liquid phase separation (LLPS), may influence the spatiotemporal organization of oncogenic and immune signaling networks (61). Although LLPS has been increasingly implicated in cancer biology, its contribution to the coordination of circadian, microbiome-derived, and immune signals in CRC remains untested. It should therefore be treated as a future mechanistic question rather than as an established component of the TIS framework.

## 7 Conclusion

In conclusion, the circadian-microbiome-immune axis provides a framework for examining how temporal regulation, microbial ecology, epithelial barrier function, and antitumor immunity may intersect in CRC. In pMMR/MSS tumors, current evidence is derived predominantly from preclinical models, with only limited clinical associations. The TIS should therefore be regarded as a hypothesis-generating conceptual model rather than an experimentally validated biological entity. It proposes that circadian disruption may contribute to barrier dysfunction, microbial and metabolic remodeling, and temporally varying accumulation of immunosuppressive myeloid cells, but the causal sequence, reproducibility across models, and relevance to human CRC remain uncertain. Interindividual differences in circadian phase, host genetics, environmental exposures, microbiome composition, and treatment context are likely to influence these relationships (16, 18). Future studies should prioritize time-resolved human sampling, functional validation of candidate microbial and metabolic mediators, and prospective biomarker-informed trials before the TIS framework is used to guide therapeutic scheduling. Thus, the principal value of the TIS lies in generating experimentally testable questions and organizing current evidence rather than defining an established mechanism of immunotherapy resistance.

## Data Availability

The original contributions presented in the study are included in the article/supplementary material. Further inquiries can be directed to the corresponding author.
